# Relationships between mitochondrial content and bioenergetics with obesity, body composition and fat distribution in healthy older adults

**DOI:** 10.1186/s40608-015-0070-4

**Published:** 2015-10-06

**Authors:** Manish S. Bharadwaj, Daniel J. Tyrrell, Iris Leng, Jamehl L. Demons, Mary F. Lyles, J. Jeffrey Carr, Barbara J. Nicklas, Anthony J. A. Molina

**Affiliations:** Sticht Center on Aging & Department of Internal Medicine, Section on Gerontology and Geriatrics, Wake Forest School of Medicine, Winston-Salem, NC 27157 USA; Department of Biostatistical Sciences, Wake Forest School of Medicine, Winston-Salem, NC 27157 USA; Department of Radiology and Radiological Sciences, Vanderbilt University Medical Center, Nashville, TN 37203 USA

**Keywords:** Mitochondria, Muscle, Aging, Adiposity, Obesity

## Abstract

**Background:**

Mitochondrial function declines with age; however, the relationship between adiposity and mitochondrial function among older adults is unclear. This study examined relationships between skeletal muscle mitochondrial content and electron transport chain complex 2 driven respiration with whole body and thigh composition, body fat distribution, and insulin sensitivity in older adults.

**Methods:**

25 healthy, sedentary, weight-stable men (*N* = 13) and women (*N* = 12) >65 years of age, with a BMI range of 18-35 kg/m^2^, participated in this study. Vastus lateralis biopsies were analyzed for citrate synthase (CS) activity and succinate mediated respiration of isolated mitochondria. Whole body and thigh composition were measured by DXA and CT. HOMA-IR was calculated using fasting glucose and insulin as an estimate of insulin sensitivity.

**Results:**

Similar to reports in middle-aged adults, skeletal muscle CS activity was negatively correlated with BMI (R = −0.43) in our cohort of older adults. Higher total and thigh adiposity were correlated with lower CS activity independent of BMI (R = −0.50 and −0.71 respectively). Maximal complex 2 driven mitochondrial respiration was negatively correlated with lower body adiposity in males (R = −0.66). In this cohort of non-diabetic older adults, both HOMA-IR and insulin were positively correlated with CS activity when controlling for BMI (R = 0.57 and 0.66 respectively).

**Conclusions:**

Adiposity and body composition are correlated with skeletal muscle mitochondrial content and electron transport chain function in healthy, sedentary, community dwelling, older adults. Specific relationships of mitochondrial bioenergetics with gender and insulin sensitivity are also apparent.

**Trial registration:**

ClinicalTrials.gov identifier NCT01049698

**Electronic supplementary material:**

The online version of this article (doi:10.1186/s40608-015-0070-4) contains supplementary material, which is available to authorized users.

## Background

The increasing prevalence of obesity among older adults has led to growing concerns about numerous chronic health conditions in this population. Total adiposity, as well as fat deposition within specific anatomical regions (e.g., body fat distribution), is associated with impairments in physical function and higher incidence of disability in older age [1–6]. In addition, age-associated changes in body composition and fat distribution contribute to the increased prevalence of cardiometabolic disorders in this age group [7, 8]. Most notably, central adiposity is strongly associated with insulin resistance and its deleterious effects on glucose homeostasis even in older age [9]. On the other hand, higher lower body adiposity, commonly referred to as the “pear-shaped” body fat distribution, is more common in women and is associated with lower cardiometabolic risk [10, 11]. Thus, age and gender related differences in total and regional adiposity are likely underlying determinants of several deleterious changes in metabolic and physiologic function common with aging.

Aging-related bioenergetic decline is highlighted by mitochondrial impairments and reduced cellular energy supply. Skeletal muscle mitochondria are particularly sensitive to aging-related decline and have been reported to decrease ATP production, maximal respiratory capacity, mtDNA content, enzyme activity, and biogenesis with older age [12–17]. However, contributions of age-associated gains in body fat and shifts in body composition and fat distribution to declining mitochondrial function in older adults are not known. Studies in middle-aged adults have reported that differences in body composition and insulin resistance have profound effects on skeletal muscle mitochondrial metabolism [18–27]. Among the bioenergetic differences linked to obesity and insulin resistance in these previous studies, oxidative phosphorylation (OXPHOS) activity and mitochondrial content have been reported to be significantly reduced.

To date, few studies in elderly humans have investigated the links between properties of skeletal muscle mitochondria and obesity, body composition, body fat distribution, and insulin sensitivity. In the present study, we examined skeletal muscle bioenergetics in two ways; 1) citrate synthase (CS) activity, and 2) OXPHOS activity assessed by respirometric profiling of isolated organelles. CS activity is reported to be a reliable biomarker of mitochondrial content in skeletal muscle, as opposed to other measures such as mtDNA copy number [28]. Analysis of respiratory control in isolated mitochondria permits direct investigation of oxidative phosphorylation and has served as a standard readout of mitochondrial function for decades [29, 30]. Our purpose was to determine whether these mitochondrial readouts are associated with obesity, body composition, body fat distribution and insulin sensitivity in older men and women.

## Methods

### Participants

This study included 25 sedentary men (*n* = 13) and women (*n* = 12) recruited, using general media advertisements and mass mailings, to participate in a clinical trial of resistance training with or without dietary-induced weight loss. The assessments reported here were conducted at baseline, prior to intervention. All participants were older (65-79 years), sedentary (no regular exercise for >20 mins, 2 d/wk), with body mass index (BMI) between 18–35 kg/m2, in good health, weight stable for prior six weeks, had normal cognitive function, used no walking aids, and did not have uncontrolled diabetes or hypertension, abnormal liver or kidney function, or cancer requiring treatment in the past 2 years. Detailed inclusion and exclusion criteria have been previously reported [31, 32]. The study was approved by the Wake Forest School of Medicine Institutional Review Board and all participants provided written, informed consent to participate.

### Skeletal muscle biopsy

Skeletal muscle tissue was obtained from the thigh muscle, specifically the *vastus lateralis* of the quadriceps, in the early morning after an overnight fast. Muscle was obtained using the percutaneous needle biopsy technique with a University College Hospital needle under local anesthesia with 1 %.

lidocaine as previously described [33]. Participants were asked to refrain from taking aspirin, prescription, or over-the-counter, nonsteroidal anti-inflammatory drugs, or other compounds that may affect bleeding, platelets, or bruising for the week prior to the biopsy, and were asked to refrain from any strenuous activity for at least 36 h prior to the biopsy.

### Citrate synthase assay

Skeletal muscle (5 mg) was homogenized in 100 μl cold CelLytic MT (Sigma; St. Louis, MO) at pH 7.4 and protease inhibitor cocktail (Sigma, P8340). The homogenized sample was centrifuged at 12,000 g for 10 min and the supernatant containing the protein collected. 8 μg protein, as determined by BCA protein assay (Pierce; Rockford IL), was utilized per reaction in a final volume of 200 μl (Sigma, CS0720) at room temperature. In addition to protein, the reaction mixture contained 1X assay buffer, 300 μM acetyl CoA and 100 μM 5,5’-Dithiobis-(2-nitrobenzoic acid. The reaction was started by adding 500 μM Oxaloacetate. CS activity was measured by continuous spectrophotometric rate determination at 412 nm, according to manufacturer instructions. Each sample was run in triplicate.

### Mitochondrial isolation

Mitochondria were isolated as previously described [33]. For each participant, ~50 mg of tissue was minced into small pieces and resuspended in Chappell-Perry Buffer I (CP I) containing Nagarse. After incubation with Nagarse for 5 min at room temperature, homogenization was performed using a Bio Gen series Model PRO200 homogenizer (Pro-Scientific, Inc., Oxford, CT) equipped with a 5 mm PRO quick connect generator probe. The homogenized tissue was washed with an equal volume of CP I and 2X volume of CP II buffer by centrifuging at 600 g, 4 °C, 10 min (Eppendorf centrifuge 5804R, Eppendorf AG, Hamburg, Germany). The resulting supernatant was filtered through cheese cloth and the filtrate centrifuged at 10,000 g, 4 °C, 10 min using a Beckman ultracentrifuge, Model J2-21 M Induction drive centrifuge (Beckman-Coulter, Inc., Brea, CA) to obtain a mitochondrial pellet. The pellet was further washed first with CP II and then with CP I buffer. Finally, the pellet was suspended in mitochondrial assay buffer (MAS: sucrose 35 mM, mannitol 110 mM, KH_2_PO4 2.5 mM, MgCl_2_ 2.5 mM, HEPES 1.0 mM, EGTA 0.5 mM, Fatty-acid free BSA 0.10 %, pH 7.4) prior to respirometry. A typical yield of ~500 μg of purified mitochondria was obtained per 50 mg sample.

### Mitochondrial respirometry

All respirometry assays were performed using an XF24–3 Extracellular Flux Analyzer (Seahorse Bioscience, Billerica, MA). The procedure was previously described by Rogers et. al. and focused on respiration driven solely by complex 2 [34]. During study protocol optimization, respiration driven by complex 1 using pyruvate/malate was compared with respiration driven by complex 2 using succinate/rotenone. Complex 2 driven respiration was consistently higher, similar to previous reports [34]. In order to ensure adequate sample size and standardization, all respirometric analyses of isolated mitochondria reported in this study were performed using succinate and rotenone. Compounds were prepared in 1X MAS at 10X the final concentration required for the assay. Final concentrations of compounds were as follows: 2 mM Adenosine 5’ –diphosphate (ADP), 2 μM oligomycin, 6 μM Carbonyl cyanide 4-(trifluoromethoxy) phenylhydrazone (FCCP), and 2 μM antimycin A. 5 μg of the mitochondrial suspension was added each well and the plate centrifuged at 2000 g, 4° C, for 20 min to ensure attachment. After centrifugation, 450 ml of 1X MAS containing succinate (10 mM) and rotenone (2 μM) was gently added to each well and the experiment initiated. The respirometric assay was performed at 37 °C. Primary outcomes were: maximal State 3 respiration, initiated with ADP; and State 4o, induced by the inhibition of ATP synthase by the addition of oligomycin. An average respiratory control ratio (RCR, State3/State 4o) of 4.5 +/− 1.3 in our studies indicate that mitochondria isolated using our protocol were viable.

### Fasting insulin and insulin sensitivity index

Blood samples were collected in EDTA-treated vacutainer tubes via venipuncture in the early morning after an overnight fast, just prior to muscle biopsy. The samples were centrifuged at 4 °C for 20 min and plasma was separated and used to determine fasting plasma insulin (FPI) and fasting plasma glucose (FPG) levels. Insulin was determined using an automated immunoanalyzer (IMMULITE; Siemens Corporation, Washington DC). Insulin sensitivity was estimated using HOMA-IR calculated as (FPI × FPG)/22.5, as previously reported [35, 36].

### Body composition and fat distribution

Total fat and lean mass, and percent body fat, were measured by dual-energy x-ray absorptiometry (DXA, Hologic Delphi QDR) by a certified technician. Whole body scans were acquired with the participant supine and aligned with the scanner table as prescribed by the manufacturer. A measure of lower body adiposity (leg-to-total fat mass) was calculated as fat mass in both legs divided by total body fat mass. Waist (minimal circumference) and hip (maximal gluteal protuberance) were measured in triplicate and averaged, and waist-to-hip ratio (WHR) was calculated. Thigh muscle and adipose tissue composition was measured on a 64 slice CT scanner (LightSpeed PlusTM, General Electric Medical Systems, Milwaukee) located in the WFSM Center for Biomolecular Imaging. Thigh scans were conducted at 120 KVp, 350 mA, 10 mm helical with a pitch of 11.25 mm/rotation and a gantry speed of 0.8 s. Measurements were performed on set of slices covering 50 mm of distance from the head to foot (z-axis) of the participant with the volume centered at the junction of the proximal and middle third of the femur as measured from the scout topogram. The volume of muscle (lean) and adipose tissue was segmented and measured using the GE Healthcare, Advantage Windows 4.2 Volume Viewer (Waukesha, WI).

### Statistical analysis

Distributions of all variables were examined before any further analysis. Square root transformation of total lean mass, HOMA-IR, and insulin data was performed in order to normalize distribution. All other variables were normally distributed as assessed using Shapiro-Wilk tests. Potential differences between men and women with regard to demographics, obesity, adiposity, insulin sensitivity, and mitochondrial parameters were analyzed using unpaired two-tailed Student’s t-test (GraphPad Software, San Diego, CA). Associations between CS activity and respiration, and obesity and adiposity variables were assessed using Pearson correlation coefficients (R) for all participants and further adjusted for BMI alone, both BMI and gender; and BMI, gender, and age together. Correlation coefficients stratified by gender (and adjusted for BMI or both BMI and age) were also calculated. To examine associations between CS activity and mitochondrial respiration, and insulin sensitivity, similar correlation analyses as described above were performed (SPSS v22; Armonk, NY). Based on two-sided tests and a significance level of 0.05; with 25 participants total, we are able to detect a correlation of 0.55 or higher with a power of 82 %. For the analyses stratified by gender, with sample sizes of 13 men and 12 women, we are able to detect a correlation of 0.74 and 0.72 respectively, with a power of 81 %.

## Results

### Participant characteristics

Body composition, body fat distribution, insulin and glucose, and bioenergetic profiles are presented in Table 1 for all participants and split by gender. As expected, waist circumference, WHR, total lean mass and thigh muscle volume were higher in males, while total percent fat, and total and subcutaneous thigh fat volumes were higher in females. Women also had a higher ratio of leg-to-total fat mass, indicative of greater lower body adiposity (pear-shaped). No significant gender differences were observed for estimated insulin resistance (HOMA-IR).

**Table 1:** Demographics, Body Composition, Insulin, Glucose, and Mitochondrial Function of all Participants and Separated by Gender

	All	Female	Male	
	Mean ± SD	Mean ± SD	Mean ± SD	*P*-value2
Age (years)	69.2 ± 3.7	69.2 ± 3.7	69.2 ± 3.8	0.97
BMI (kg/m2)	27.7 ± 5.1	26.3 ± 6.1	28.9 ± 3.8	0.22
Waist Circumference (cm)	90.7 ± 16.1	83.4 ± 18.0	97.4 ± 11.0	0.03
Waist/Hip ratio	0.9 ± 0.1	0.8 ± 0.2	0.9 ± 0.1	0.15
Total Fat Mass(kg)	28.6 ± 8.9	28.8 ± 10.9	28.3 ± 6.9	0.88
Total Lean Mass1 (kg)	7.14 ± 0.8	6.5 ± 0.5	7.8 ± 0.4	0.00
Percent Fat Mass	35.4 ± 6.9	39.5 ± 6.3	31.3 ± 4.8	0.00
Leg Fat Mass to Total Fat Mass ratio	0.3 ± 0.05	0.37 ± 0.05	0.30 ± 0.04	0.00
Total Thigh Fat (cm3)	647.0 ± 237.1	751.9 ± 256.5	550.2 ± 175.9	0.03
Thigh Subcutaneous Fat (cm3)	623.7 ± 226.4	727.5 ± 241.5	528.0 ± 168.4	0.02
Thigh Intermuscular Fat (cm3)	23.3 ± 15.9	24.4 ± 18.4	22.2 ± 13.9	0.74
Total Thigh Muscle (cm3)	648.4 ± 153.5	517.4 ± 86.0	769.4 ± 85.3	0.00
Thigh Intermuscular Fat/Total Thigh Fat Ratio	0.04 ± 0.02	0.03 ± 0.01	0.04 ± 0.02	0.27
HOMA-IR1	1.5 ± 0.5	1.3 ± 0.6	1.6 ± 0.5	0.12
Insulin (mIU/L)^a^	3.1 ± 1.0	2.7 ± 1.1	3.3 ± 0.9	0.14
Glucose (mmol/L)	5.1 ± 0.5	5.0 ± 0.4	5.3 ± 0.6	0.07
State 3 OCR (pmol/min/5 μg)	499.7 ± 200.3	459.5 ± 230.0	536.8 ± 169.2	0.35
State 4o OCR (pmol/min/5 μg)	123.1 ± 68.5	120.1 ± 68.5	125.8 ± 71.1	0.84
State 3u OCR (pmol/min/5 μg)	556.6 ± 315.2	487.5 ± 344.9	620.3 ± 283.8	0.30
Respiratory Control Ratio (RCR)	4.5 ± 1.3	4.0 ± 0.6	5.0 ± 1.6	0.06
Citrate Synthase Activity (μmol/min/mg)	175.1 ± 77.1	161.3 ± 82.4	190.2 ± 71.7	0.38

*BMI* body mass index, *OCR* oxygen consumption rate, *RCR* respiratory control ratio

^a^Variables were square-root transformed to achieve normal distribution, 2 Independent samples t-test between females (*N* = 12) and males (*N* = 11-13)

### Associations of citrate synthase activity with body composition and body fat distribution

**Fig. 1:**
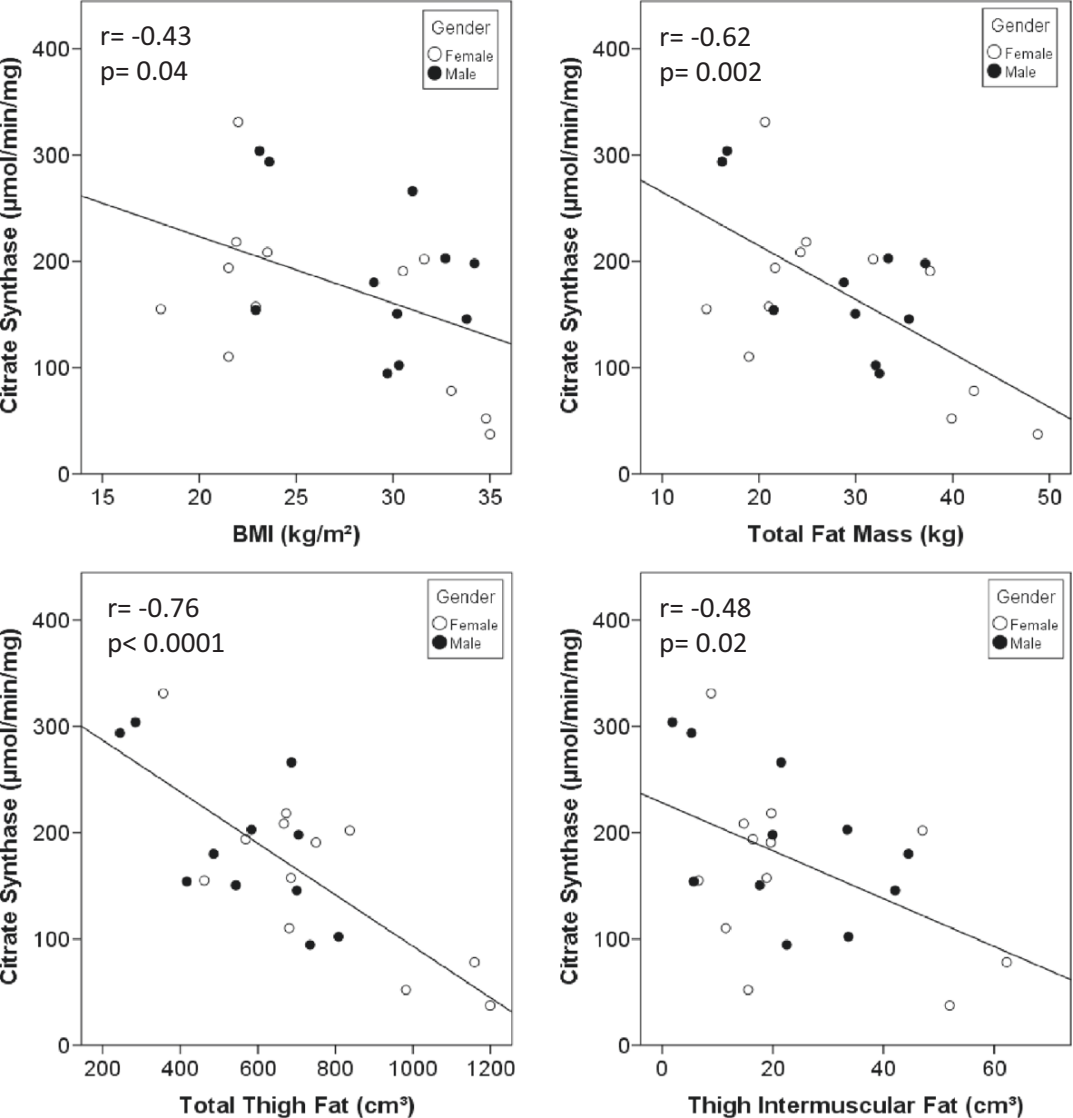
Regression analysis comparing Vastus lateralis mitochondrial content, measured as citrate synthase enzyme activity with representative body composition variables (BMI, DXA total fat mass, CT total thigh fat volume, and intermuscular thigh fat volume). Relationships between citrate synthase enzyme activity and adiposity variables.

The correlation coefficients of CS activity with BMI, whole body and thigh composition, and body fat distribution are presented in Table 2. Additionally, Fig. 1 presents scatterplots with regression lines depicting the relationship of CS activity with BMI, total fat mass, total thigh fat volume, and intermuscular thigh fat volume. Additional file 1: Figure S1 presents scatterplots with regression lines depicting the relationship of CS activity with percent total fat and subcutaneous thigh fat. We report correlations with males and females combined and separated. For combined data, partial correlations with adjustments for BMI, BMI + gender, and BMI + gender + age are reported. For gender separated data, adjustment for BMI and BMI + age is also presented. In men and women combined, skeletal muscle CS activity correlated negatively with obesity measured by BMI (R = −0.43, *P* = 0.039), and with measures of adiposity (total fat mass (R = −0.62, *P* = 0.002), percent fat (R = −0.62, *P* = 0.002); and total (R = −0.76, *P* = 0.0001), subcutaneous (R = −0.76, *P* = 0.0001), and intermuscular thigh fat volumes (R = −0.48, *P* = 0.021)). Except for intermuscular thigh fat volume, these relationships were independent of BMI, gender, and age. After adjustment for BMI, there were positive correlations between CS activity and lean mass (R = 0.43, *P* = 0.039) and thigh muscle volume (R = 0.53, *P* = 0.014) in both men and women. The contribution of BMI in CS activity correlations with total fat mass and percent fat, were different between men and women. Namely, controlling for BMI in women diminished the correlation between CS activity and total body fat (R = −0.59, *P* = 0.02 and R = −0.18, *P* = 0.60, respectively). We also observed a significant negative correlation between CS activity and leg-to-total fat ratio in both men and women after adjustment for BMI (R = −0.64, *P* = 0.002), indicating that higher lower body adiposity is negatively correlated with CS activity.

**Table 2:** Citrate Synthase Activity Correlation Coefficients with Obesity and Total and Thigh Body Composition and Body Fat Distribution for All Participants and Split by Gender

	Both Genders				Female			Males		
	Pearson	Partial (BMI)	Partial (BMI + gender)	Partial (BMI + gender + age)	Pearson	Partial (BMI)	Partial (BMI + age)	Pearson	Partial (BMI)	Partial (BMI + age)
BMI (kg/m2)	−0.43*	n/a	n/a	n/a	−0.57	n/a	n/a	−0.41	n/a	n/a
Waist Circumference (cm)	−0.30	0.20	0.07	0.21	−0.44	0.19	0.06	−0.40	−0.40	0.06
Waist/Hip ratio	−0.07	0.15	0.10	0.17	−0.11	0.10	−0.03	−0.24	0.29	0.45
Total Fat Mass (kg)	−0.62**	−0.50*	−0.41	−0.41	−0.59*	−0.18	−0.16	−0.68*	−0.88**	−0.87*
Total Lean Mass^a^ (kg)	−0.01	0.43*	0.37	0.35	−0.32	0.41	0.46	−0.08	0.34	0.17
Percent Fat Mass	−0.62**	−0.48*	−0.47*	−0.47*	−0.57	−0.15	−0.16	−0.83**	−0.88**	−0.84**
Leg Fat Mass to Total Fat Mass ratio	−0.161	−0.64**	−0.59**	−0.60**	0.01	−0.75**	−0.76*	−0.31	−0.46	−0.53
Thigh Fat Volume (cm3)	−0.76***	−0.71***	−0.80***	−0.71***	−0.80**	−0.77**	−0.81**	−0.68*	−0.84**	−0.56
Thigh Subcutaneous Fat (cm3)	−0.76***	−0.72***	−0.81***	−0.72***	−0.82***	−0.80**	−0.83**	−0.67*	−0.82**	−0.55
Thigh Intermuscular Fat (cm3)	−0.48*	−0.24	−0.15	−0.11	−0.46	−0.07	−0.30	−0.51	−0.29	0.10
Total Thigh Muscle (cm3)	0.20	0.53*	0.52*	0.53*	−0.18	0.56	0.56	0.37	0.53	0.30
Thigh Intermuscular Fat/Total Thigh Fat Ratio	−0.18	0.10	0.07	0.11	−0.13	0.28	0.17	−0.33	−0.06	0.22

*BMI* body mass index

^a^Variables were square-root-transformed to achieve normal distribution

* = *p* ≤ 0.05, ** = *p* ≤ 0.01, *** = *p* ≤ 0.001

Females: *N* = 12; Males: *N* = 10-11

### Association of mitochondrial respiration with body composition and body fat distribution

**Fig. 2:**
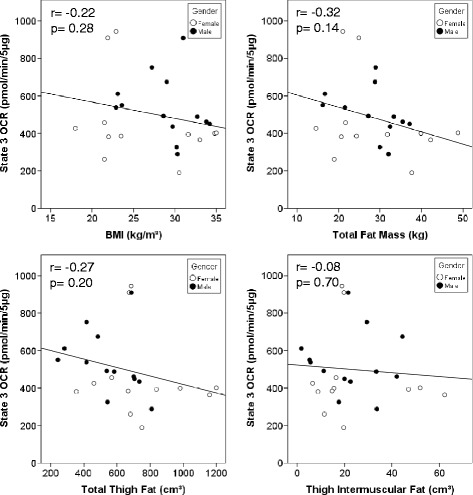
Regression analysis comparing Vastus lateralis mitochondrial state 3 respiration, measured as OCR in response to saturating ADP, with representative body composition variables (BMI, DXA total fat mass, CT total thigh fat volume, and intermuscular thigh fat volume) Relationships between state 3 respiration, BMI, and adiposity variables

The correlation coefficients of complex 2 driven mitochondrial respiratory state 3 (maximal) with BMI, total body and thigh composition, and body fat distribution are presented in Table 3. Additionally, Fig. 2 presents scatterplots with regression lines depicting the specific correlations with BMI, total fat mass, thigh fat volume, and intermuscular thigh fat volume. In males, maximal state 3 OCR significantly correlates with lower body adiposity (R = −0.66, *P* = 0.019). Analysis of state 4o respiration (Table 4) indicates that there are no significant correlations with obesity and body composition. The correlation coefficients of RCR (state 3/state 4o) with BMI, total body and thigh composition, and body fat distribution are presented in Additional file 2: Table S1. RCR is positively correlated with blood glucose (R = 0.48, *P* = 0.016). Gender separated data indicate a significant negative correlation with total thigh fat (R = −0.69, *P* = 0.019) and subcutaneous thigh fat (R = −0.71, *P* = 0.015) after adjusting for BMI in women. Likewise, a strong positive correlation is seen with HOMA-IR and insulin after adjusting for BMI (R = 0.63, *P* = 0.038, and R = 0.64, *P* = 0.036 respectively) in women.

**Table 3:** State 3 Respiration Correlation Coefficients with Obesity and Total and Thigh Body Composition and Body Fat Distribution for All Participants and Split by Gender

	Both Genders				Female			Male		
	Pearson	Partial (BMI)	Partial (BMI + gender)	Partial (BMI + gender + age)	Pearson	Partial (BMI)	Partial (BMI + age)	Pearson	Partial (BMI)	Partial (BMI + age)
BMI (kg/m2)	−0.22	n/a	n/a	n/a	−0.32	n/a	n/a	−0.26	n/a	n/a
Waist Circumference (cm)	−0.01	0.33	0.27	0.49*	−0.17	0.27	0.39	−0.06	0.29	0.76**
Waist/Hip ratio	0.19	0.29	0.28	0.45*	0.16	0.28	0.48	0.16	0.41	0.48
Total Fat Mass (kg)	−0.32	−0.14	0.00	0.10	−0.28	0.08	0.09	−0.41	−0.26	0.17
Total Lean Mass^a^ (kg)	0.061	0.25	0.14	0.17	−0.26	0.03	0.06	0.04	0.40	0.36
Percent Fat Mass	−0.35	−0.20	−0.04	0.04	−0.23	0.16	0.16	−0.50	−0.47	−0.13
Leg Fat Mass to Total Fat Mass ratio	0.01	−0.05	0.00	−0.10	0.37	0.26	0.26	−0.66*	−0.68*	−0.71*
Total Thigh Fat (cm3)	−0.29	−0.12	0.07	0.14	−0.13	0.33	0.32	−0.38	−0.59	−0.11
Thigh Subcutaneous Fat (cm3)	−0.30	−0.14	0.04	0.11	−0.13	0.32	0.32	−0.39	−0.62	−0.15
Thigh Intermuscular Fat (cm3)	−0.08	0.17	0.25	0.40	−0.08	0.24	0.29	−0.44	0.33	0.60
Total Thigh Muscle (cm3)	0.12	0.14	−0.07	−0.03	−0.49	−0.42	−0.36	0.28	0.37	0.14
Thigh Intermuscular Fat/Total Thigh Fat Ratio	0.15	0.27	0.26	0.37	−0.01	0.18	0.31	0.25	0.56	0.57

*BMI* body mass index

^a^Variables were square-root-transformed to achieve normal distribution

* = *p* ≤ 0.05, ** = *p* ≤ 0.01,

Females: *N* = 12; Males: *N* = 12-13

**Table 4:** State 4o Respiration Correlation Coefficients with Obesity and Total and Thigh Body Composition and Body Fat Distribution for All Participants and Split by Gender

	Both Genders				Female			Male		
	Pearson	Partial (BMI)	Partial (BMI + gender)	Partial (BMI + gender + age)	Pearson	Partial (BMI)	Partial (BMI + age)	Pearson	Partial (BMI)	Partial (BMI + age)
BMI (kg/m2)	−0.24	n/a	n/a	n/a	−0.20	n/a	n/a	−0.35	n/a	n/a
Waist Circumference (cm)	−0.09	0.19	0.24	0.43*	−0.06	0.28	0.38	−0.20	0.24	0.58
Waist/Hip ratio	0.19	0.29	0.28	0.36	0.27	0.38	0.47	−0.17	−0.08	0.11
Total Fat Mass (kg)	−0.28	0.11	0.12	0.13	−0.16	0.13	0.12	−0.52	0.08	0.19
Total Lean Mass^a^ (kg)	−0.13	0.05	0.20	0.14	−0.16	0.02	0.07	−0.12	0.40	0.21
Percent Fat Mass	−0.18	0.04	0.00	0.07	−0.10	0.20	0.16	−0.55	−0.23	0.03
Leg Fat Mass to Total Fat Mass ratio	0.15	−0.00	−0.02	−0.06	0.35	0.29	0.28	−0.26	−0.38	−0.39
Thigh Fat Volume (cm3)	−0.12	0.15	0.19	0.29	0.03	0.46	0.42	−0.31	−0.27	0.23
Thigh Subcutaneous Fat (cm3)	−0.12	0.14	0.17	0.26	0.03	0.45	0.12	−0.31	−0.30	0.19
Thigh Intermuscular Fat (cm3)	−0.09	0.24	0.23	0.31	0.02	0.25	0.33	−0.22	0.29	0.37
Total Thigh Muscle (cm3)	−0.01	−0.04	−0.01	0.00	−0.40	−0.40	−0.33	0.22	0.26	0.03
Thigh Intermuscular Fat/Total Thigh Fat Ratio	−0.03	0.19	0.20	0.18	0.03	0.19	0.29	−0.07	0.43	0.27

*BMI* body mass index

^a^Variables were square-root-transformed to achieve normal distribution

* = *p* ≤ 0.05

Females: *N* = 13, Males: *N* = 12-13

### Relationships with estimated insulin sensitivity

The correlation coefficients of CS activity, state 3, and state 4o OCR with estimated insulin resistance (HOMA-IR), fasting insulin, and glucose are presented in Table 5. CS activity correlated positively with fasting insulin (R = 0.66, *P* = 0.0001) and HOMA-IR (R = 0.57, *P* = 0.002) after controlling for BMI (Additional file 3: Figure S2). Gender separated data indicate similar results between men and women.

**Table 5:** Correlation Coefficients for Mitochondrial Measures and Glucose/Insulin for All Participants and Split by Gender

	Both Genders				Female			Male		
	Pearson	Partial (BMI)	Partial (BMI + gender)	Partial (BMI + gender + age)	Pearson	Partial (BMI)	Partial (BMI + age)	Pearson	Partial (BMI)	Partial (BMI + age)
Citrate Synthase Associations										
HOMA-IR^a^	0.21	0.57**	0.55*	0.58**	0.08	0.62*	0.61	0.26	0.46	0.53
Insulin (mIU/L)^a^	0.20	0.66***	0.63**	0.60**	0.09	0.71*	0.63	0.25	0.49	0.51
Glucose (mmol/L)	0.19	0.31	0.25	0.34	0.09	0.58	0.61	0.18	0.05	0.44
State 3 OCR Associations										
HOMA-IR^a^	−0.19	−0.27	−0.32	−0.12	−0.53	−0.45	−0.39	−0.08	−0.05	0.20
Insulin (mIU/L)^a^	−0.19	−0.24	−0.31	−0.13	−0.51	−0.43	−0.39	0.08	0.00	0.19
Glucose (mmol/L)	−0.07	−0.22	−0.30	−0.02	−0.35	−0.22	−0.17	−0.05	−0.58	0.14
State 4o OCR Associations										
HOMA-IR^a^	−0.27	−0.39	−0.39	−0.16	−0.54	−0.53	−0.45	−0.06	−0.21	0.10
Insulin (mIU/L)^a^	−0.27	−0.36	−0.36	−0.16	−0.52	−0.52	−0.46	−0.05	−0.15	0.10
Glucose (mmol/L)	−0.21	−0.38	−0.38	−0.07	−0.33	−0.26	−0.18	−0.20	−0.72*	0.01

*BMI* body mass index, *HOMA-IR* homeostatic model assessment of insulin resistance, *OCR* oxygen consumption rate

^a^Variables were square-root-transformed to achieve normal distribution

* = *p* ≤ 0.05, ** = *p* ≤ 0.01, *** = *p* ≤ 0.001

Females: *N* = 12, Males: *N* = 11-13

## Discussion

Age related bioenergetic decline has been associated with numerous age related diseases and is thought to be mediated by numerous factors such as mitochondrial DNA mutations and oxidative stress [37, 38]. Among the physiological changes that occur with aging, there is growing concern that increased adiposity and changes in body fat distribution lead not only to increased cardiometabolic risk, but also increased risk for disability. Recent studies have highlighted the significance of skeletal muscle mitochondrial function in the physical ability of older adults [31, 39–41]. However, little is known about the relationship of obesity, adiposity, and mitochondrial bioenergetics in this age group. In this manuscript, we present evidence that bioenergetic deficits among older adults are related to differences in adiposity and body fat distribution. Our data indicate that older men and women with a higher BMI as well as total and thigh adiposity have lower skeletal muscle (*vastus lateralis*) CS activity, a biomarker of mitochondrial content [28]. In particular, thigh adiposity was strongly associated with low mitochondrial mass, independent of BMI. The apposition of thigh adipose tissue to *vastus lateralis* may contribute to the strength of this correlation. A possible interpretation of our findings is that changes in body composition related to aging can contribute to age-related bioenergetic decline. Alternatively, alterations in mitochondrial function may play a role in altering body fat distribution with aging. Longitudinal studies are required in order to understand the cause and effect relationship of bioenergetic decline and age-related changes in adiposity and body fat distribution.

In addition to age, gender is known to underlie differences in regional fat deposition and body composition. While women generally have more body fat than men, the pear-shaped body fat distribution is thought to contribute to lower cardiometabolic risk [10, 11, 42]. It has been suggested that gluteal-femoral adipose tissues may represent a safe lipid reservoir or may directly regulate systemic metabolism via release of metabolic products or adipokines [11]. In this study, we analyzed the relationship of adiposity and skeletal muscle mitochondria across genders, as well as in men and women alone. We observe that thigh fat volume, particularly in the subcutaneous depot, is strongly associated with low skeletal muscle mitochondrial content regardless of gender. When controlling for BMI, a higher ratio of leg fat to total fat was associated with lower skeletal muscle mitochondrial content.

Skeletal muscle bioenergetic decline has been previously associated with diabetes and insulin resistance [21, 22, 43, 44]. Both blood insulin and HOMA-IR positively correlated with mitochondrial mass when controlling for BMI. A potential interpretation of this result is that in healthy older adults, CS activity may increase in relation to mild insulin resistance. Our cohort of healthy, non-diabetic, older adults had an average HOMA-IR of 1.5 ± 0.5. It is conceivable that higher levels of insulin resistance are required before deleterious effects on mitochondrial content are observed. Future studies across a wider range of insulin sensitivities are required in order to determine at what point mitochondrial content is decreased.

A recent study reported that state 3 respiration of skeletal muscle is significantly reduced in obese individuals compared to lean controls even when normalizing for mitochondrial content [26]. Furthermore, they report that weight loss with bariatric surgery was able to improve mitochondrial function one year after surgery. In this study, we examined intrinsic mitochondrial function by profiling mitochondrial respiration driven by electron transport chain (ETC.) complex 2 in isolated organelles. In separate studies of obese and older adults, increased activity of complexes 2–4 (assessed by succinate oxidase activity) has been reported with exercise intervention [14, 45]. In the present study, the correlation between complex 2 driven state 3 OCR, a measure of maximal mitochondrial respiration, with adiposity, expressed as percent fat measured by DXA, did not reach statistical significance. Examination of gender separated data suggests that these correlations, while not statistically significant, are stronger in men compared to women. Interestingly, lower body adiposity in men was significantly associated with lower state 3 respiration. Taken together, these data suggest that lower body adiposity may be more detrimental to *vastus lateralis* mitochondrial content as well as intrinsic mitochondrial function in men compared to women. On the other hand, our data also indicate that the association of insulin resistance and intrinsic mitochondrial function is stronger in women, although not reaching statistical significance in this study. These data suggest that ETC. dysfunction, evident with mild insulin resistance, may be related to multiple mitochondrial changes others have reported in diabetic patients [20–22, 44]. It should be noted that various mitochondrial isolation techniques can result in altered bioenergetic function, such as significantly increased RCR compared with permeabilized myofibers accompanied by significantly reduced time to mitochondrial permeability transition pore opening and greater reactive oxygen species production [46]. The use of mitochondrial isolation methodologies is a potential limitation to our study and must be considered in the interpretation of our results. Future studies should be undertaken in order to more comprehensively examine respiration driven by complex 1 and β-oxidation, in addition to complex 2. Such studies will likely require significantly larger amounts of biopsied tissue if performed in isolated mitochondria. A potentially powerful approach would be to perform respirometric analysis of permeabilized muscle fibers. Smaller sample requirements can allow for more comprehensive analysis of mitochondrial function. Moreover potential damage associated with mitochondrial isolation procedures can be avoided [46]. In addition, mitochondrial function can be affected by a number of other factors; including, ROS production, calcium retention capacity, and apoptotic susceptibility. These can be examined using methodologies similar to those that have been described for respirometry with combined fluorometry [47–50].

## Conclusion

Changes in adiposity and body fat distribution associated with aging are thought to play an important role in the cardiometabolic health and physical ability of older adults. While it is apparent that adiposity and body fat distribution negatively impact skeletal muscle mitochondrial bioenergetics in middle aged adults, relatively little is known about the relationship of adiposity on mitochondrial function in older adults, a population that has been shown to exhibit systemic bioenergetic decline. This study is the first to report that differences in total adiposity as well as body fat distribution among healthy, community dwelling, older adults are related to skeletal muscle mitochondrial bioenergetics as reported by both citrate synthase activity and intrinsic electron transport chain function driven by complex 2. Specific relationships of mitochondrial bioenergetics with gender and insulin sensitivity were apparent and reveal important areas for future research.

## Additional files

Additional file 1: Figure S1. Regression analysis comparing Vastus lateralis mitochondrial content, measured as citrate synthase enzyme activity with percent fat and subcutaneous thigh fat mass. (ODP 37 kb) Additional file 2: Table S1. Respiratory Control Ratio Correlation Coefficients with Obesity and Total and Thigh Body Composition and Body Fat Distribution for All Participants and Split by Gender (ODP 32 kb) Additional file 3: Figure S2. Regression analysis comparing Vastus lateralis mitochondrial content, measured as citrate synthase enzyme activity with HOMA IR. (PPTX 72 kb)

## Abbreviations

BMI: Body mass index
CP: Chappel-Perry
CS: Citrate synthase
CT: Computed tomography
DXA: Dual energy X-ray absorptiometry
ETC.: Electron transport chain
FCCP: Carbonyl cyanide 4-(trifluoromethoxy) phenylhydrazone
FPG: Fasting plasma glucose
FPI: Fasting plasma insulin
HOMA-IR: Homeostatic model assessment-estimated insulin resistance
MAS: Mitochondria assay solution
mtDNA: Mitochondrial DNA
OCR: Oxygen consumption rate
RCR: Respiratory control ratio
WHR: Waist-to-hip ratio
