# Supplementary material for: Relationships between mitochondrial content and bioenergetics with obesity, body composition and fat distribution in healthy older adults
Source: BMC Obes. 2015 Oct 6;2:40. doi: 10.1186/s40608-015-0070-4 (PMC4594906; doi:10.1186/s40608-015-0070-4)
Supplement: Additional file 3: Figure S2. — Regression analysis comparing Vastus lateralis mitochondrial content, measured as citrate synthase enzyme activity with HOMA IR. (PPTX 72 kb) [file 40608_2015_70_MOESM3_ESM.pptx]

## Slide 1
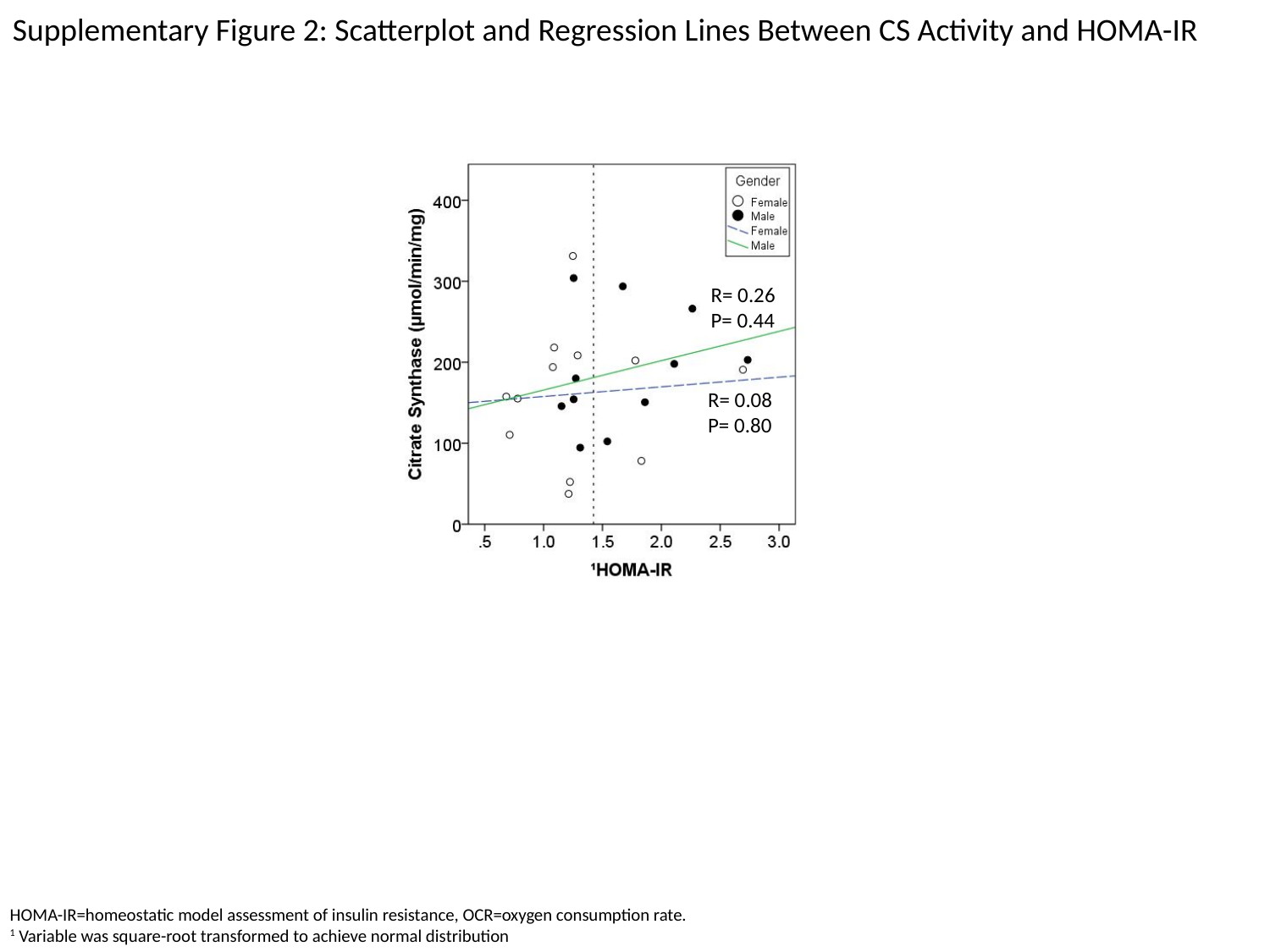

Supplementary Figure 2: Scatterplot and Regression Lines Between CS Activity and HOMA-IR
R= 0.26
P= 0.44
R= 0.08
P= 0.80
HOMA-IR=homeostatic model assessment of insulin resistance, OCR=oxygen consumption rate.
1 Variable was square-root transformed to achieve normal distribution
